# The history of head transplantation: a review

**DOI:** 10.1007/s00701-016-2984-0

**Published:** 2016-10-14

**Authors:** Nayan Lamba, Daniel Holsgrove, Marike L. Broekman

**Affiliations:** 1 Cushing Neurosurgery Outcomes Center, Department of Neurosurgery, Brigham and Women’s Hospital, Harvard Medical School, Boston, MA USA; 2 Department of Neurosurgery, Greater Manchester Service Centre, Salford Royal Hospital, Salford, UK; 3 Department of Neurosurgery, University Medical Center Utrecht, Heidelberglaan 100, 3584 CX Utrecht, The Netherlands

**Keywords:** Head transplantation, Spinal cord fusion, Brain transplant, Cephalosomatic anastomosis

## Abstract

**Background:**

Since the turn of the last century, the prospect of head transplantation has captured the imagination of scientists and the general public. Recently, head transplant has regained attention in popular media, as neurosurgeons have proposed performing this procedure in 2017. Given the potential impact of such a procedure, we were interested in learning the history of the technical hurdles that need to be overcome, and determine if it is even technically possible to perform such a procedure on humans today.

**Method:**

We conducted a historical review of available literature on the technical challenges and developments of head transplantation. The many social, psychological, ethical, religious, cultural, and legal questions of head transplantation were beyond the scope of this review.

**Results:**

Our historical review identified the following important technical considerations related to performing a head transplant: maintenance of blood flow to an isolated brain via vessel anastomosis; availability of immunosuppressive agents; spinal anastomosis and fusion following cord transfection; pain control in the recipient. Several animal studies have demonstrated success in maintaining recipient cerebral perfusion and achieving immunosuppression. However, there is currently sparse evidence in favor of successful spinal anastomosis and fusion after transection. While recent publications by an Italian group offer novel approaches to this challenge, research on this topic has been sparse and hinges on procedures performed in animal models in the 1970s. How transferrable these older methods are to the human nervous system is unclear and warrants further exploration.

**Conclusions:**

Our review identified several important considerations related to performing a viable head transplantation. Besides the technical challenges that remain, there are important ethical issues to consider, such as exploitation of vulnerable patients and informed consent. Thus, besides the remaining technical challenges, these ethical issues will also need to be addressed before moving these studies to the clinic.

## Introduction

Ever since the beginning of the last century, with advances in medicine and specifically transplantation, the prospect of head transplant has captured the imagination of scientists and the general public. Recently, head transplant has regained attention in the popular media, as neurosurgeons have proposed to perform this procedure in the near future.

Given the potential impact of such a procedure, we were interested in learning the history of various technical hurdles that need to be overcome, and based on these studies, determine if it is even technically possible to perform such a procedure on humans today. To this aim, we present a historical review of the available literature on the technical challenges and developments of head transplantation. The many social, psychological, ethical, religious, cultural, and legal questions of head transplantation are beyond the scope of this review.

## Methods

### Electronic search strategy

We searched PubMed and EMBASE using the following keywords: “head transplant,” “brain transplant,” or “cephalosomatic anastomosis” (Tables 1 and 2). The last search was conducted in June 2016.

**Table 1:** PubMed search string

head transplant[tiab] OR brain transplant[tiab] OR (cephalosomatic[All Fields] AND anastomosis[All Fields])
Results: 42 hits, 7 June 2016

**Table 2:** EMBASE search string

‘head’/exp OR head AND transplant.tw OR ‘brain’/exp OR brain AND transplant.tw OR cephalosomatic AND (‘anastomosis’/exp OR anastomosis)
Results: 3 hits, 7 June 2016

### Eligibility criteria and study selection

Titles and abstracts of references obtained from the literature search were screened. We excluded articles written in languages other than English (*n* = 3). Inclusion criteria consisted of any study that described head or brain transplantation. After title and abstracting screening, remaining articles were read in full. One author of the team performed full-text screening of the articles. One senior author verified the final included articles. Disagreements were solved by discussion. This review was restricted to published data and was not limited by date of publication.

Additional papers were included after hand-searching of the bibliographies of the included papers.

## Results

**Fig. 1:**
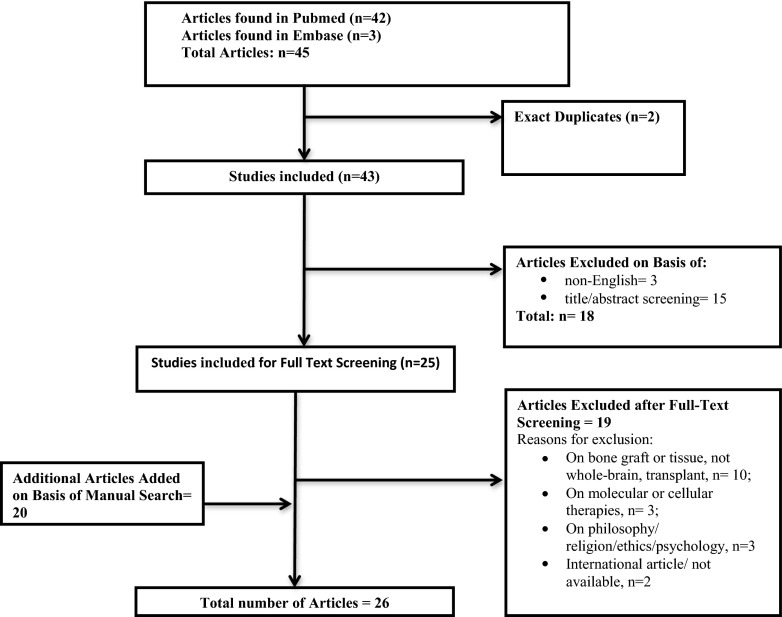
Study selection process for the identified articles

After a thorough search of the literature on the history of head transplantation, we identified 30 papers describing five technical challenges relevant for head transplantation (Fig. 1).

### Beginning of head transplantation and vessel anastomosis

Ever since the early 1900s, people have discussed the possibility of head transplantation [17, 18]. However, transplantation surgery at that time faced many challenges.

One of the main challenges in transplant surgery was reliable vessel anastomosis.

The challenge faced by vascular surgeons was how to cut and repair an injured vessel and subsequently restore blood flow without interrupting circulation. Bone, silver and gold, and absorbable material grafts were in use in the early 1900s, but these materials gave uncertain and variable results in patients [18].

It was French surgeon, Dr. Alexis Carrel, who changed these results by using a more reliable method of suturing severed vessels back together: he used fine needles and extremely thin threads as suture and enlarged the severed vessel opening using three retaining sutures to form a triangular shape. His method proved effective in protecting against postoperative hemorrhages and embolisms, as well as in preventing strictures at the site of the suture [18]. Carrel was able to successfully implement this technique to vessel reconstruction and whole organ transplantation (mainly in procedures involving the thyroid and kidney) [18].

In 1908, Carrel and American physiologist, Dr. Charles Guthrie, performed the first dog head transplantation. They attached one dog’s head onto another dog’s neck, connecting arteries in such a way that blood flowed first to the decapitated head and then to the recipient head. The decapitated head was without blood flow for about 20 min, and while the dog demonstrated aural, visual, and cutaneous reflex movements early after the procedure, its condition soon deteriorated and it was euthanized after a few hours [9, 11].

While their work in head transplantation was not particularly successful, Carrel and Guthrie made significant contributions to the transplant field’s understanding of vessel anastomosis. In 1912, they were awarded the Nobel Prize in Physiology and Medicine for their work on limb and organ transplantation [17].

Another milestone in the history of head transplantation was reached in the 1950s due to the work of Soviet scientist and surgeon Dr. Vladmir Demikhov. Like his predecessors, Carrel and Guthrie, Demikhov made notable contributions to the field of transplant surgery, especially thoracic surgery. He improved upon the methods available at the time for maintaining vascular supply during organ transplantation and was able to perform the first successful coronary bypass surgery in dogs in 1953. Four dogs survived for more than 2 years after this surgery [14].

**Fig. 2:**
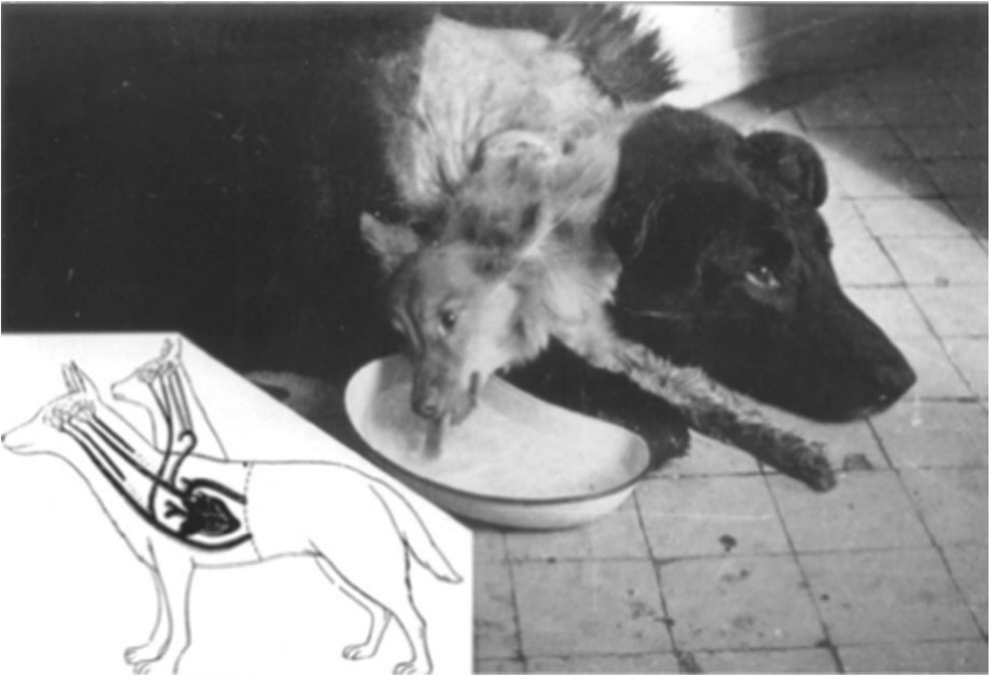
Two-headed dog from Demikhov’s experiment. Reprinted from Konstantinov [14]

In 1954, Demikhov also attempted a canine head transplant (Fig. 2). Demikhov’s dogs demonstrated more functional capacity than Guthrie and Carrel’s dogs and were able to move, see, and lap up water [9]. A step-by-step documentation of Demikhov’s protocol published in 1959 reveals how his team carefully preserved the blood supply to the lung and hearts of the donor dog:

First they made an incision at the base of the large dog’s neck, exposing the jugular vein, the aorta and a segment of the spinal column. Next they drilled two holes through the bony part of one vertebra and threaded two plastic strings, one red and one white, through each of the holes… Then he and Demikhov, deftly wielding the scalpel, needle and thread, proceeded with infinite pains to expose the small blood vessels, drawing a tight knot of thread around each one in turn as they carved gradually deeper into Shavka’s vitals. Finally Demikhov severed the spinal column [16].

Thus, even though the rest of its body had been amputated from this dog, its head and forepaws still retained and used the lungs and heart [16, 24]. During the third phase of the transplantation, the main blood vessels of this dog’s head were connected with the corresponding vessels of the host dog [16]. The longest that any dog survived this surgery was 29 days, also longer than Guthrie’s and Carrel’s dogs, but most died within a few days [9].

This limited survival was mainly due to an immune response of the recipient to the donor, and the next major challenge of head transplantation involved the lack of immunosuppressive agents available at the time [1, 9]. Without effective ways to manage the immune-rejection reactions between the donor and recipient, Demikhov’s work could not be considered in the clinical realm for use in humans [1]. The issue of immunosuppression will be addressed below.

In 1965, Robert White, an American neurosurgeon, also attempted head transplantation.

His goal was to perform a brain transplant onto an isolated body, contrary to Guthrie and Demikhov, who transplanted the entire upper body of a dog, and not just the isolated brain [1].

This required him to establish different perfusion techniques than his predecessors.

In fact, maintaining blood flow to an isolated brain was the biggest challenge for White. He created vascular loops to preserve anastomoses between the internal maxillary and internal carotid arteries of the donor dog. This system was referred to as “auto-perfusion,” in that it allowed for the brain to be perfused by its own carotid system even after being severed at the second cervical vertebral body [25]. Next, the brain was positioned between the jugular vein and carotid artery of the recipient [15]. Using these perfusion methods, White was able to successfully graft six canine brains to the cervical vasculature of six large recipient dogs. The dogs survived between 6 h and 2 days [25].

By continuous electroencephalogram (EEG) monitoring, White monitored the viability of the transplanted cerebral tissue and compared the activity of the transplant brain to the recipient brain. Moreover, using an implantable recording module, he also kept track of the metabolic state of the brains via measurements of oxygen and glucose consumption and demonstrated that the transplanted brains were in a high-performance metabolic state after surgery, another indication of functional success of the transplants [25].

Through his auto-perfusion protocol and subsequent tracking of brain function, White demonstrated the short-term feasibility of a pure brain, as opposed to upper body, transplant.

White expressed an interest in using these methods to develop a model system whereby he could induce tumor formation, meningitis, or encephalitis in the brains being transplanted and subsequently test whether the normal recipient animal’s circulating blood could restore functionality to injured brains [15].

**Fig. 3:**
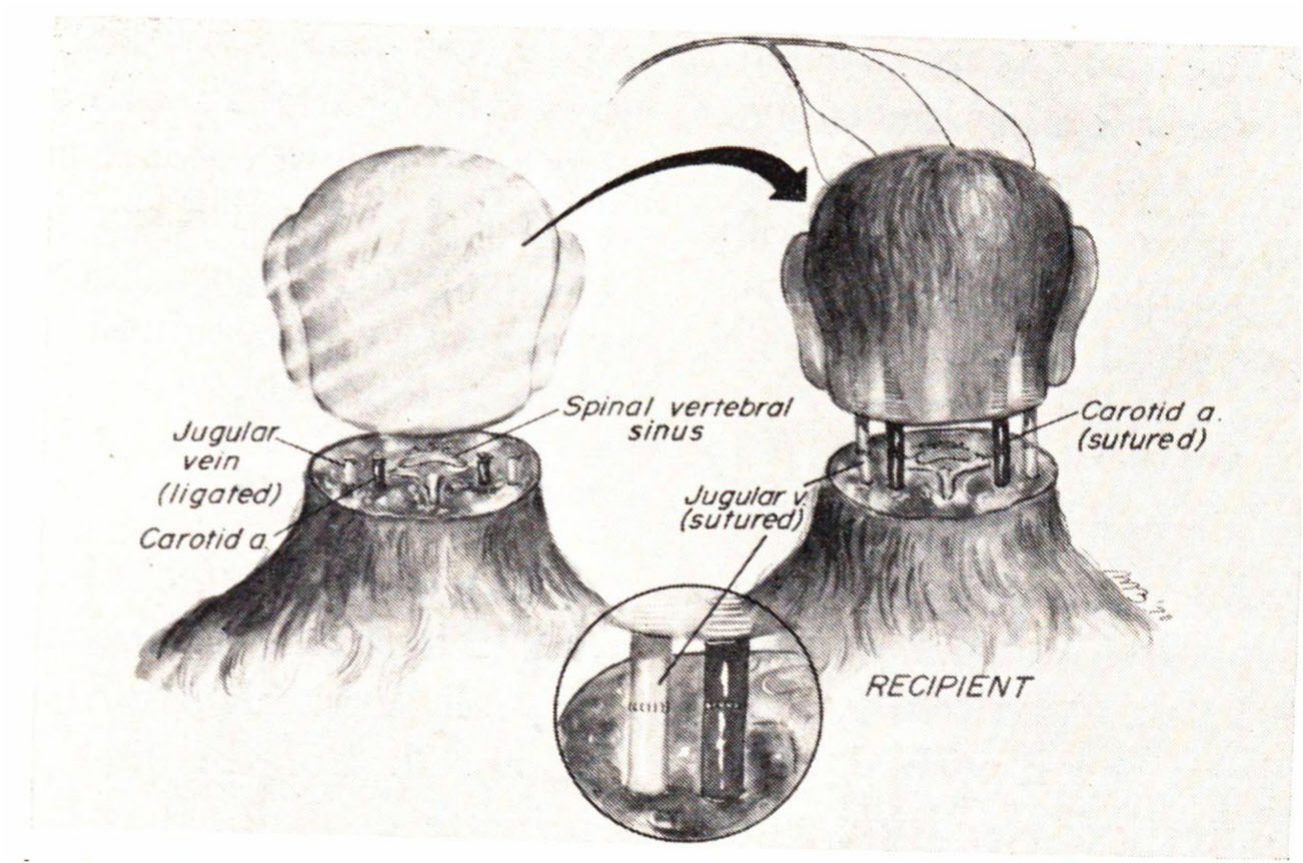
From White’s original paper, showing the isolated primate cephalon transplanted to the isolated monkey body via direct suture of the carotid and jugular vessels. *Reprinted from White 1971* [26]

In 1970, White performed the first cephalic exchange transplantation in primates (Fig. 3). He performed four cephalosomatic associations between isolated monkey heads and isolated monkey bodies, employing direct suture of the carotid and jugular veins. Cervical laminectomy was performed at the level of the fourth through sixth cerebral vertebrae. Due to the subsequent spinal shock and hypotension following transection, catecholamine infusion was started and mechanical pulmonary support initiated and maintained throughout the remainder of the experiment [15]. Three to four hours after surgery, each cephalon was able to chew, swallow food, track with eyes, and bite if orally stimulated [26]. Moreover, through EEG monitoring, White demonstrated that these cephalons exhibited a characteristic awake pattern [26].

Despite the aforementioned advances, revascularization in the cephalon remained a challenge and survival ranged between 6 and 36 h [26]. Due to constriction that developed in the jugular vein at the suture line, venous return from the head was impeded [26]. The direct suture was, therefore, not successful enough to allow for unimpeded blood flow, and the cephalons required continuous infusion with heparin; this eventually led to blood loss and was a limiting factor in the longevity of this experiment [26]. Moreover, White acknowledged that the cervical spine transection was another limitation of his methods, as it necessitated the implementation of continuous respiratory support for the animal [15]. Spinal transection and cephalon revascularization and ischemia will be addressed below.

**Fig. 4:**
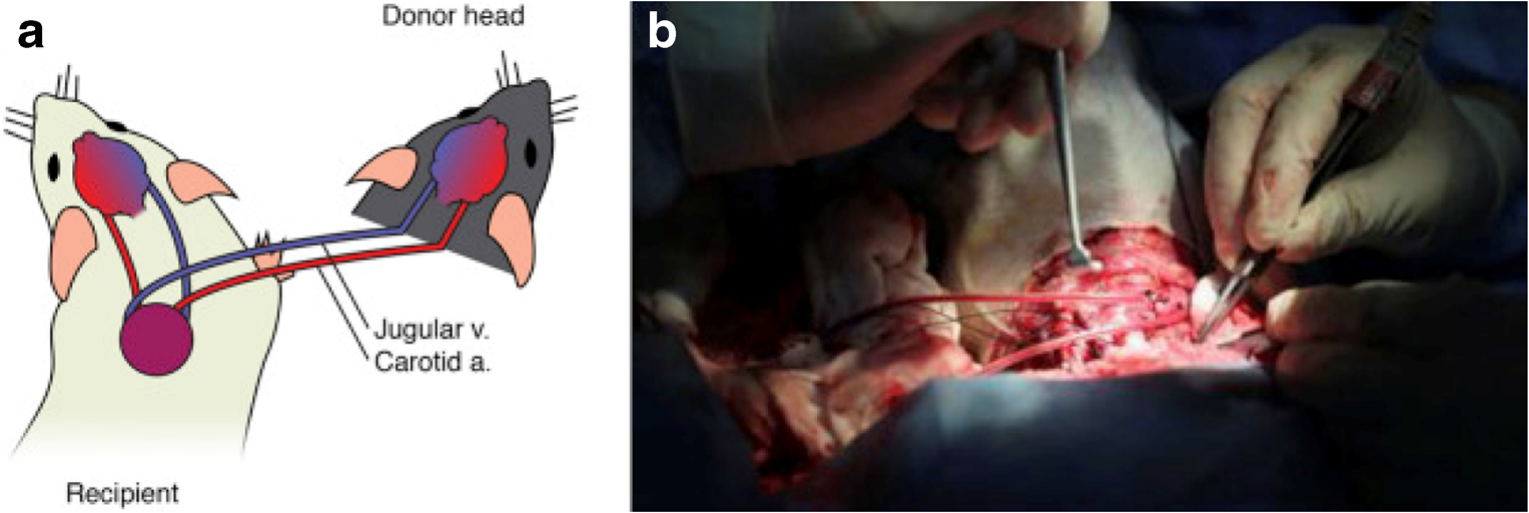
(**a**) Jugular-carotid cross circulation in the mouse model of head transplantation. (**b**) The first monkey head transplantation with cross circulation enacted. *Reprinted from Ren 2016* [21]

Further modification of optimal vessel anastomoses occurred years later in 2015 by the Chinese surgeon, Xiao-Ping Ren, [22]. In contrast to the previously described direct anastomosis, he utilized a method in which only one carotid artery and the contralateral jugular vein were cut, allowing the intact carotid artery and jugular vein to continuously perfuse the donor head throughout the procedure (Fig. 4) [22]. Using this protocol for head-body transplants in mice, he was able to maintain the blood pressure of the mice above 100/60 mm Hg during the entire procedure. Moreover, EEG recordings from both the donor and recipient heads after surgery demonstrated normal electrical activity [22]. By cutting only one carotid and jugular vessel, Ren’s method minimized trauma to the recipient, prevented development of ischemia, and also allowed for intact brain function [26]. Over half of his mice survived for periods longer than 24 h, with the longest survival being 6 months [22]. While White’s dog and primate head transplants had demonstrated short-term success, in part due to complications related to clotting, heparinization, and ischemia, Ren’s revascularization protocol allowed for longer-term survival in mice [22].

### Immunosuppression

In 1912, when Guthrie and Carrel were presented with their Nobel Prize, it was acknowledged that the work they performed “had no application in man,” and that organs transplanted from one to another will degenerate in their new owners. While progress had been made in understanding maintenance of perfusion during transplant surgery, issues like immunosuppression remained prominent.

In the 1950s and 60s, the face of transplant surgery changed. The challenge that had been preventing the long-term success of experiments by Carrel, Guthrie, and Demikhov had been addressed. Immunosuppressive agents like azathioprine, 6-mercaptopurine, and corticosteroids were discovered, and teams of physicians around the world began to perform human kidney and heart transplants [1].

When White was performing his transplant experiments in the 1960s and 70s, immunosuppressive agents were now available. White utilized these drugs in his monkey head transplantations and was able to prevent the occurrence of hyper-rejection reactions in primates, as demonstrated by histology of the brain tissue after their death [26]. However, this came at the cost of extremely high doses of immunosuppression [1, 25]. In fact, the high doses of immunosuppressive agents required to prevent rejection actually contributed to the death of White’s monkeys 9 days after transplant.

Early failure of White’s monkeys was at least in part due to the toxicity of the high dose of immunosuppressive agents. In addition, grafts involving the skin posed an additional challenge. Due to the highly immunogenic nature of skin tissue, transplants involving hands and faces were not responsive to the immunosuppressive agents of the time [1]. It was not until 1999 that a combination of immunosuppressive agents was discovered that was effective in preventing skin rejection without toxicity if used in the correct dosages [13]. In particular, lower dose combinations of tacrolimus and mycophenolate mofetil were believed to prevent tissue rejection without causing systemic toxicity in the recipient, representing the ideal balance for skin transplant patients [13]. Indeed, in 2006, the first human face allograft proved successful with the use of thymoglobulin, tacrolimus, mycophenolate mofetil, and prednisone [8]. Whether or not the efficacy and safety of these newer immunosuppressive agents will hold up in human head transplants, however, remains to be determined.

Ren [22] has highlighted that it has been particularly difficult to study immune-rejection in head transplant patients because of their short-term survival. However, he believes that his cross-circulation protocol, which extends survival in head transplant patients, will allow for careful study of immune-mediated rejection reactions and lead to an understanding of optimal immunosuppressive regimens for head transplantation.

### Spinal anastomosis

An additional aspect of head transplantation that White and his predecessors struggled with was the fusion of the donor-recipient spinal cords. In his study of head transplantation in primates, White noted that spinal cord severing during the procedure would ultimately result in the requirement for continuous respiratory support after transplant [26]. How to successfully fuse the donor and recipient spinal cords and allow for gain of motor function was not addressed until the recent experiments of Ren and Italian neurosurgeon, Dr. Sergio Canavero.

In 2014, Ren offered an alternative to the traditional head transplantation method of transecting the spinal cord. Before Ren, transection occurred at the C3/C4 level and therefore did not preserve the brainstem of the donor [20]. Independent breathing and circulation were lost, and life-support machines were needed. This was a severe limitation White acknowledged in his primate experiments [26]. In contrast, by performing mouse head transplants that maintained the integrity of the donor brainstem, Ren’s donor animal could breathe on its own after transplantation. Ren’s utilization of a transection site above the brainstem, thereby offered promise for independent breathing of the donor and longer survival times compared with the traditional C3/C4 transections performed by White and those before him [20].

Around the same time as Ren, Canavero also put forth his own head transplant protocol, “HEAVEN,” or Head Anastomosis Venture [6]. GEMINI is a component of “HEAVEN” that sets it apart from prior models of head transplantation, addressing this very issue of spinal cord integrity.

Canavero’s spinal anastomosis protocol calls for acute, tightly controlled spinal cord transection, unlike what occurs during traumatic spinal cord injury [4]. He posits that a controlled transection will allow for tissue integrity to be maintained and subsequent recovery and fusion to occur [6]. Specifically, he highlights how his protocol will exploit a secondary pathway in the brain, the cortico-truncoreticulopropriospinal pathway [4]. This “propriospinal interneuronal system,” first discovered by Charles Sherrington in the early 1900s, is a gray-matter system of intrinsic fibers that forms a network of connections between spinal cord segments. When the primary, corticospinal tract is injured, the severed corticospinal tract axons can form new connections via these propriospinal neurons (PNs) [4]. Numerous animal studies have demonstrated that the propriospinal neurons act as an “anatomic bridge” and allow for motor function and recovery in animals with a damaged corticospinal tract [6]. In GEMINI, Canavero plans to perform a highly controlled transection of the spinal cord that inflicts minimal damage to the gray matter [4]. His theory is that the maintenance of these grey matter PNs will allow for functional recovery to be achieved after spinal cord transection in humans undergoing a head transplant [4].

A recent review acknowledges a significant role for propiospinal neurons in recovery after spinal cord injury [10]. However, this regenerative response of PNs comes from studies in numerous animal models, including cats, rats, and mice, which have different spinal cord circuitry and regenerative capacities than humans [10]. For example, the authors of this review describe a classic experiment in which cats underwent spinal cord transection at the lumbar level. Remarkably, these spinalized cats were able to recover weight-bearing hind limb stepping that closely resembled the normal feline walking pattern [10]. The authors went on to acknowledge, however, that while many similarities do exist across species in terms of structural and functional reorganization of sublesional spinal circuits, cats are particularly plastic in that they are the only mammal species that can recover walking without extrinsic stimulation of the lumbar spinal cord [10]. Whether the same regenerative principles that have been observed in animal models will hold after human spinal cord transection therefore remains to be elucidated.

It should be noted, however, that similar experiments have been attempted on primates, as well. In the aforementioned review, the authors describe one experiment in which primates underwent incomplete cervical spinal cord injury and, following propriospinal neuron-mediated reorganization, were able to recover significant reaching and digit movements [10]. Still, the authors acknowledge that the precise mechanism by which PNs lead to “re-wiring” after injury remains unclear [10]. They emphasize that a great deal of research aimed at understanding the molecular basis and fundamental physiology of re-wiring is warranted before the therapeutic potential of propriospinal circuitry can be truly harnessed [10].

### Fusogens

The recent development of “fusogens” has also contributed to progress in the field of spinal anastomosis and recovery. “Fusogens” refer to polymers, like polyethylene glycol (PEG), poloxamers, and poloxamines, that have the ability to fuse the membranes of cells together [10]. In 2004, a team led by Dr. Richard Borgens at Purdue University treated paraplegic dogs with PEG injections within 72 h after their spinal cord injury and found that more than half of the treated dogs were able to walk within 2 weeks of treatment [2]. Of note, Borgens himself stated that while the results of his study demonstrated that PEG could offer a clear benefit to dogs with acute spinal cord injury, there are significant differences between canine and human spinal cords that must be addressed before this therapy could reach the human clinical realm [2]. Moreover, Borgens induced spinal cord injury in his model via a constant displacement compression/crush technique [7]. This type of injury is notably different from the type of transection that would be performed during head transplantation, and therefore limits the applicability of Borgens’ success with PEG in the procedure of head transplantation.

In their 2012 review, Cho and Borgens [7] described their success in applying PEG nanoparticles to guinea pigs with spinal cord injury. They performed in vivo testing of PEG application and measured physiological recovery via somatosensory evoked potentials (SSEP). Again, they were able to demonstrate several notable features of PEG as a fusogen, including its specificity to injured sites, “sealing” of disrupted membranes, reduction in generation of reactive oxygen species and lipid peroxidation, and, as mentioned, functional recovery as measured by recovery of SSEP conduction [7]. However, as before, the guinea pigs in Cho and Borgens’ study did not experience spinal cord transection; instead the animals they applied PEG to had been inflicted with a spinal cord compression injury [7]. Therefore, the success that Cho and Borgens demonstrated with respect to the use of PEG after compression injury is not directly generalizable to the procedure of spinal cord transection, as would occur in head transplantation.

In his GEMINI protocol, Canavero discussed the aforementioned work of Borgens and Cho in dogs and guinea pigs, and described his plan to use PEG to reconstitute neural membranes after human spinal cord transection [4, 6]. In addition to the fact that these animals underwent a compression injury and did not have their spinal cords transected, another limitation of the studies that Canavero relied on is that they focus on the efficacy of PEG as a fusogen in rats, dogs, or guinea pigs, with sparse evidence for its use as a spinal cord fusogen in humans [6]. While a phase-one safety trial using PEG on uninjured human volunteers has been successfully completed, further exploration and testing may be warranted before PEG can be applied to injured humans [4, 7].

In a recent editorial about his GEMINI protocol, Canavero proposed that in addition to fusogens, electrical stimulation can also be used to accelerate recovery of the neurons severed during spinal cord transection [5]. He cited the successful clinical application of spinal cord stimulation (SCS) in humans with spinal cord injury [5]. However, one of the studies he referenced describes three patients with chronic, incomplete spinal cord injury who were ambulating with assistive devices before the stimulation [12]. He also referenced SCS efficacy in stroke and neural injury rehabilitation. However, these patient injuries do not mirror those that will follow the acute spinal cord transection that would occur during a human head transplantation. Therefore, as with fusogens, further study should be performed to understand how safe and effective SCS might be after spinal cord transection in particular.

### Revascularization, neuroprotection, cerebral ischemia

As described in the section above on vessel anastomosis, Ren et al.’s 2015 execution of head transplantations in mice was successful in maintaining blood flow to the recipient brain during the entire procedure [20]. The key in this experiment was that the brain of the recipient animal was perfused by the blood flow from the carotid artery of the donor animal via a connection to one of its own carotid arteries (Fig. 2) [21]. Furthermore, blood was drained back into the donor internal jugular vein via the recipient’s own internal jugular vein [21]. This jugular-carotid cross circulation approach allowed for uninterrupted perfusion of the recipient’s brain tissue and thereby prevented the development of cerebral ischemia [21]. When head transplantation is performed in humans, however, Ren et al. [21] anticipate that there will be a short period of cerebrovascular arrest due to technical constraints, such as the physical distance between the gurneys.

Induced hypothermia is a widely used neuroprotective treatment utilized in patients with cardiac arrest, stroke, and hypoxic ischemic encephalopathy [21]. In White et al.’s [26] early experiments in monkeys, he implemented deep hypothermia (<25 °C) to protect the transplanted brain during ischemic periods of the procedure. However, in Ren’s model described above, he was able to successfully carry out a head transplantation with induction of only moderate hypothermia in the mice (29-33 °C), a less aggressive temperature than White et al. had to use [21].

With regards to hypothermia, Ren et al. [21] acknowledged that the optimal time for cooling has not yet been established. However, they did not think it would be of concern in their team’s proposed cephalosomatic anastomosis (CSA) protocol, since the donor’s brain-dead body would not undergo hypothermia, and the recipient’s body (while it may suffer the complications of hypothermia such as bradycardias, hypotension, and thrombosis) would be of no consequence since its body would ultimately be discarded [21].

In addition to hypothermia optimization, there has also been research exploring pharmacologic agents to preserve brain function following procedural ischemia [21]. For example, perftoran, a gas-transferring fluorinated organic compound, has been used as a blood substitute during hypothermia and was demonstrated to offer substantial neuroprotection and maintenance of cerebral oxygenation in a randomized trial of 50 patients [19, 21]. Hydrogen sulfide may also be a candidate for use in CSA due to its potential role as a neuroprotectant gas [21]. However, thus far, no clinical studies have demonstrated its effectiveness in patients with cerebral ischemia [21].

Thus, while Ren et al.’s cross circulation and moderate hypothermia protocol has proven effective in avoiding cerebral ischemia in mice head transplantation, further research into its application on humans, as well as the additional safety offered by neuroprotective agents, needs to be performed.

### Pain control

An issue that was not addressed by early head transplant researchers, likely because they performed experiments on mice and primates, was that of pain. Canavero et al. [3] published a paper this year that acknowledges the development of pain as a possible postoperative complication following head transplantation. They suggest that central neuropathic pain (CCP) could be dealt with through a selective lesion in the subparietal white matter that targets the sensory component of chronic pain [3]. Through high-intensity focused ultrasound, they believe that they can minimize bleeding, engage in real-time monitoring, and avoid collateral damage [3]. This procedure is still very experimental, with no clinical studies showing that this would actually relieve symptoms of CCP. Indeed, as the author points out, further research is warranted before potential application for chronic post transplant CCP [3].

### Future considerations

Head transplantation experiments have been conducted for over a century, starting with dogs in 1908 and extending to primate models in the 1970s. Over the past decades, improvements in vascular surgery, immunosuppressive agents, and spinal cord recovery have in some reignited enthusiasm for potential human head transplants.

Multiple press releases confirm that the author of most of the recent studies on head transplantation has the intention to move forward with the first human head transplantation in December 2017. He was quoted as saying, “We have already proved everything we had to prove” [23].

Our historical review identifies several important considerations related to performing a viable head transplantation, including maintenance of central nervous system perfusion, spinal anastomosis and fusion, and pain control (Table 3). Recent publications differ from earlier publications as they propose techniques that would allow for maintenance of spinal cord and brainstem function after transplant, essential for long-term viability and autonomy of the recipient human. However, as detailed above, recent research in this field has been sparse, dominated by a single group, and, in part, hinges on procedures performed in dogs, mice, and Rhesus monkeys in the 1970s. How transferrable the methods of Carrel, Guthrie, Demikhov, White, and coworkers are to the human nervous system is unclear and warrants elaborate further exploration before we should even consider offering humans this procedure. Thus, despite the progress made since the first experiments in 1908, some important technical challenges do remain (Table 3).

**Table 3:** The technical challenges in performing a safe and viable human head transplant

Technical challenge	Successfully overcome?	Progress to date	Remaining issues
Vessel anastomosis	Yes	• Carrel & Guthrie (1908): improved vessel suturing techniques • Demikhov (1954): maintained blood flow to lungs and heart despite spinal cord transection • White (1965): auto-perfusion of brain after transection of cervical body • Ren et al. (2015): cross-circulation protocol/prevention of brain ischemia via preservation of one donor carotid and one donor jugular vessel	
Immunosuppression	Yes	• 1950s and 1960s: development of azathioprine, 6-mercaptopurine, and corticosteroids • 1999: discovery of immunosuppressive agents that were effective in preventing skin rejection without toxicity of earlier agents	• Optimizing safety and efficacy of newer immunosuppressive agents
Spinal cord transection and anastomosis	No	• Canavero (2013): controlled spinal cord transection to maintain tissue integrity and allow for functional recovery • Ren et al. (2014): spinal cord transection below C3/C4 to allow for maintenance of brainstem and independent donor breathing	• Based on numerous animal models that do not duplicate human physiology • Weak understanding of the propriospinal circuitry thought to underlie spinal cord recovery
Spinal cord fusion	No	• Borgens and Cho (2004-2012): demonstrated success of “fusogens,” like PEG, in spinal cord recovery after injury	• Fusogen efficacy based on animal models (dogs and guinea pigs); further testing of PEG on injured humans is warranted • Injuries to which fusogens applied are not equivalent to spinal cord transection that would occur in human head transplantation
Revascularization, neuroprotection, cerebral ischemia	No	• Ren et al. (2015): moderate hypothermia with cross-circulation approach allows for minimal cerebral ischemia • Putintsev et al. (2008): demonstrated neuroprotective role of perftoran during periods of cerebral ischemia	• Human head transplantation would require period of cerebral ischemia greater than that in mice experiments due to technical obstacles during surgery • Efficacy of many potential neuroprotective pharmacologic agents has not been validated in clinical settings
Pain control	No		• Current modalities to address post-operative central neuropathic pain lack support from clinical studies; current approaches based on theory

Besides the technical challenges, there are also important ethical issues to consider before embarking on a procedure like this. These include questions like: how would a successful transplant change the meaning of human identity? While the literature and media are referring to this procedure as a head transplantation, it is truly a body transplantation, in which a person’s brain is receiving a new body. The question then becomes: how will patients who emerge from such a life-changing procedure react to having a new body to control and associate with their identity? These are complex issues that warrant further exploration. Besides the technical challenges, these issues will need to be addressed before transitioning these studies into clinical practice.
